# New Optimization Strategies on Radiation Protection in Fluoroscopy-Guided Interventional Procedures in Pediatrics

**DOI:** 10.3390/children10050883

**Published:** 2023-05-15

**Authors:** Carlos Ubeda

**Affiliations:** Departamento de Tecnología Médica, Laboratorio de Dosimetría Personal (LABODOP), Facultad de Ciencias de la Salud, Universidad de Tarapacá, Arica Casilla 7D, Chile; cubeda@cademicos.uta.cl

The term fluoroscopically guided interventional procedure describes a clinical practice in medicine, where fluoroscopic systems are used to conduct diagnostic procedures or provide image guidance for therapeutic interventional procedures performed via percutaneous or other access routes [1]. These procedures are increasingly used in pediatric patients as minimally invasive procedures that can replace more complex surgical options, especially in pediatric interventional cardiology [2].

Fluoroscopically guided interventional procedures may involve high radiation doses to patients [3]. Special attention must be paid to pediatric patients undergoing these procedures in comparison with adult patients, as children are potentially at greater risk of radiation-induced stochastic effects due to a higher radiation sensitivity of their tissues [4,5], and they have a longer lifespan in which long-term carcinogenic effects can develop [3].

Due to the above concerns, it must be a priority to avoid unnecessarily high doses to pediatric patients, applying the principles of radiological protection system, in particular in the strategies that allow radiological safety to be optimized during these procedures [5], despite the fact that modern X-ray systems come with more options to reduce doses to patients and operators, thanks to their new detectors, automatic dose management programs, etc. The basic aim of the optimization of radiological protection during a fluoroscopically guided interventional procedure is to adjust imaging parameters and institute protective measures in such a way that the required image is obtained with the lowest possible radiation dose, and net benefit is maximized [6]. Some examples of optimization strategies might be: quality assurance programs, characterization of dose and image quality of X-ray systems [7,8], quality control tests of X-ray systems, the analysis of patient dose metrics, establishment of diagnostic reference levels (DRLs) classified by ranges of weight and age, among others. Some possible concrete actions for optimization might be: reducing the radiation dose to the minimum needed (“ALARA” principle), reducing the field to the strictly necessary part of the body, avoiding unnecessary double planes, using a low-dose-rate fluoroscopy mode when possible, minimizing fluoroscopy time, using fluoroscopy only to guide devices if absolutely necessary and observe motion, using the last-image-hold image for review when possible, instead of using fluoroscopy, minimizing the number of cine series, reducing the number of personnel present in the fluoroscopically guided interventional laboratory to the minimum needed, posing careful indications, considering non-radiating alternatives if possible, etc.

This special issue will mainly show the outcomes achieved in the OPRIPALC (Optimization of Protection in Pediatric Interventional Radiology in Latin America and the Caribbean) program [9], although also articles that are framed within the optimization strategies for other types of fluoroscopically guided pediatric procedures may be accepted.

OPRIPALC was conceived as a joint response of the Pan American Health Organization and the World Health Organization, in cooperation with the International Atomic Energy Agency, to support their member states in Latin America and the Caribbean in ensuring that radiation exposures of pediatric patients are appropriate for the respective fluoroscopically guided interventional procedures [10,11]. To our knowledge, this initiative is unique worldwide.

This special issue will present articles to share the evolution, advancements, and challenges for the OPRIPALC program. Likewise, as one of the main products of this international initiative, an article on good practices for the optimization of protection and safety in fluoroscopically guided interventional pediatric procedures will be prepared. Furthermore, two systematic reviews on ranges of pediatric radiation dose indices in interventional cardiology procedures and the image quality metrics used to characterize X-ray equipment and optimize fluoroscopically guided interventional procedures will be presented. In addition, the use of DRLs will be part of this effort, showing the experience and results accumulated by the countries participating in OPRIPALC. Finally, the uses of automatic dose management systems, their advantages, and disadvantages will be discussed.

## Data Availability

Not applicable.
